# Development and Characterization of Oleogels from Avocado Oil and Monoglycerides

**DOI:** 10.3390/foods15030478

**Published:** 2026-01-30

**Authors:** Michael Moreno-Caballero, Jenny Paola Ortega-Barbosa, Liliam Alexandra Palomeque-Forero, María Cristina Lizarazo-Aparicio, Diego Miranda-Lasprilla, Diego Ballesteros-Vivas, Fabián Parada-Alfonso, Elena Ibañez-Ezequiel

**Affiliations:** 1 Food Chemistry Research Group, Universidad Nacional de Colombia, Ave Cra 30 #45-3, Bogotá 111321, Colombia; micmorenoca@unal.edu.co (M.M.-C.); jportegab@unal.edu.co (J.P.O.-B.); lapalomequef@unal.edu.co (L.A.P.-F.); fparadaa@unal.edu.co (F.P.-A.); 2 Instituto de Ciencia y Tecnología de Alimentos, Faculty of Agricultural Sciences, Universidad Nacional de Colombia, Ave Cra 30 #45-3, Bogotá 111321, Colombia; mlizarazoa@unal.edu.co; 3 Faculty of Agricultural Sciences, Universidad Nacional de Colombia, Ave Cra 30 #45-3, Bogotá 111321, Colombia; dmirandal@unal.edu.co; 4 Grupo de Investigación Alimentos, Nutrición y Salud, Departamento de Nutrición y Bioquímica, Pontificia Universidad Javeriana, Cra 7 No 40–62, Bogotá 110231, Colombia; diego_ballesteros@javeriana.edu.co; 5 Foodomics Group, Food Science Research Institute (CIAL), The Spanish National Research Council (CSIC), Nicolás Cabrera 9, Campus UAM Cantoblanco, 28049 Madrid, Spain

**Keywords:** oleogels, avocado oil, rheology, firmness, monoglycerides and oxidative stability

## Abstract

The growing demand for healthier lipid alternatives has driven interest in oleogels as promising substitutes for the conventional saturated and trans fats in foods systems. In this context, this study explores the formulation and characterization of oleogels based on avocado oil (*Persea americana var. Lorena*) and monoglycerides, as an alternative to conventional saturated fats. Hydraulic compression was used to extract the oil, and the formulation was optimized using a Box–Behnken experimental design, evaluating the effects of temperature (70–90 °C), monoglyceride concentration (4–8%), and heating time (15–45 min) on oil retention capacity (ORC) and firmness. Results showed that temperature and concentration significantly influenced ORC and firmness, while heating time had no relevant effect. The optimal formulation achieved 85.95% ORC, 1.09 N firmness, and superior oxidative stability (41.71 h vs. 10.80 h in pure oil, Rancimat test). The obtained oleogel exhibited good mechanical and thermal properties, with an elastic-dominant rheological profile and higher oxidation resistance compared to unmodified avocado oil. These findings indicate that the avocado oleogels structured with monoglycerides have potential applications in the food and cosmetic industries, although further improvements in structural stability are recommended to broaden their range of applications.

## 1. Introduction

Fat is a crucial component in the food industry, serving as an energy source and as a lipid matrix that facilitates the delivery of fat-soluble vitamins (A, D, E, and K), while also enhancing the physical, chemical, and sensory properties of food products [1,2]. Solid fats are valued for their oxidative stability, ability to extend shelf life, and contribution to desirable textures [3]. However, many of these fats, primarily composed of triglycerides, contain saturated or trans fatty acids, which have been linked to an increased risk of cardiovascular diseases, obesity, and type II diabetes [4,5,6].

Due to public health concerns, recent research has focused on developing healthier substitutes, highlighting oils rich in unsaturated fatty acids as a promising alternative [7,8]. However, replacing saturated fats with unsaturated ones presents challenges due to differences in properties such as melting point and solidification capacity. These differences negatively impact the sensory and functional characteristics of food products, leading to issues such as poor aeration in cakes [1], low melting points in chocolate [6,9], and reduced firmness and texture in spreads and baked goods [10,11].

An effective strategy to solidify unsaturated oils without altering their composition is structuring them into oleogels. Various methods have been reported, including direct dispersion, emulsion-templated, and solvent exchange techniques [12]. While indirect methods may involve solvents, complex steps, or limited scalability, direct dispersion is solvent-free, simple, and highly suitable for food applications [6,13]. In this approach, structuring agents such as monoglycerides (MGs), waxes, and polymers are dispersed in the oil, and controlled cooling promotes crystallization to form the gel network [14,15]. The properties of the oleogels depend on both the nature of the structurant and its interaction with the oil phase [16].

Among the studied structuring agents, monoglycerides (MGs) stand out for their efficient structuring ability and low cost, making them suitable for food applications [17,18]. Previous studies have shown that MG-based oleogels exhibit a melting range between 55 and 90 °C, which facilitates their crystallization during cooling and allows them to form stable three-dimensional networks. Concentrations of MGs in the range of 5–12% (*w*/*w*) are commonly employed, with higher levels generally leading to stronger gel networks due to increased crystal density and oil entrapment. Rheologically, MG-based oleogels often show storage modulus (G′) values exceeding the loss modulus (G″), low loss tangent (tan δ), and measurable yield stress [19,20,21]. These rheological features are particularly beneficial because they govern mechanical stability, spreadability, and resistance to deformation during processing and consumption, explaining the suitability of MG-based oleogels as alternatives to trans or saturated fats in baked goods, confectionery, and spreads [22,23].

Avocado oil (*Persea americana Mill.*) Var. Lorena is an attractive oil phase for oleogel development due to its unique composition and functional benefits. It contains a high proportion of unsaturated fatty acids, with oleic acid often exceeding 60%, alongside minor bioactive compounds such as tocopherols, phytosterols, and chlorophylls, which contribute to its nutritional and antioxidant properties [24]. Compared to other vegetable oils, avocado oil shows favorable oxidative stability and a distinctive profile of bioactives, supporting both its technological functionality and added value in food, cosmetic, and pharmaceutical applications [24,25]. These characteristics justify its selection as a structuring medium in the design of oleogels aimed at delivering both technological performance and health-promoting benefits.

In recent years, global avocado consumption has increased significantly, particularly in countries such as the United States, France, Germany, and the United Kingdom. As production has expanded, market prices have declined; however, a considerable proportion of harvested fruits fails to meet export quality standards, mainly due to size or visual imperfections. This generates economic losses for producers and results in underutilized raw material [25]. The valorization of non-export-grade avocado through its transformation into functional lipid ingredients, such as oleogels, offers a promising strategy to reduce waste, increase profitability, and respond to industry demands for healthier fat alternatives.

Therefore, this study aims to develop and optimize avocado oil oleogels structured with monoglycerides using a Box–Behnken experimental design. The research specifically evaluates their physicochemical properties, rheological behavior, and oxidative stability to determine their potential as healthier substitutes for conventional solid fats. Additionally, this work contributes to the valorization of surplus or non-export-grade avocado by proposing its use as a functional lipid matrix in food reformulation.

## 2. Materials and Methods

### 2.1. Plant Material

The avocado fruit (*Persea americana var. Lorena*) variety Lorena was provided by farmers from the Casanare region (eastern Colombia). The avocados were randomly harvested from various farms located in three municipalities: Monterey, Sabanalarga, and Tauramena, depending on availability during harvest seasons. After collection, the fruits were cleaned with a 5% sodium hypochlorite solution and allowed to ripen at room temperature until reaching the optimal consumption point, defined by a soft texture with Brix values between 8 and 10. The pulp of the samples was then extracted, and they were stored at −20 °C until processing. The monoglyceride used was Masemul^®^ DMG 6002, IMCD, Milan, Italy, containing a minimum of 60% monoglycerides derived from fully hydrogenated palm oil, supplied by Deltagengroup Colombia, Bogotá, Colombia. 

### 2.2. Oil Extraction

**Figure 1:**
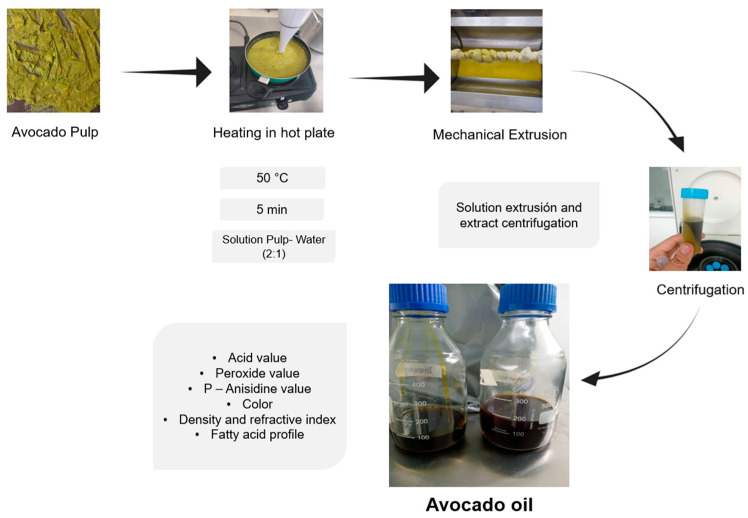
Process for obtaining avocado oil.

To obtain the oil, a methodology based on hydraulic compression was implemented [24,25]. The procedure involved an initial drying step using a heating plate (GRS–54, Hatco Corporation, Milwaukee, WI, USA), in which avocado pulp was spread in layers with a thickness of less than 8 mm, and dried at 80 °C for 4 h, until a final moisture content below 8% was reached. Subsequently, the dried pulp was subject to hydraulic compression using 0.5 mm thick fabric mesh and a hydraulic press equipped with a 61 mm piston, applying a maximum compression force of 20 tons (67.16 MPa). Figure 1 presents a diagram of the process used. The quality parameters and fatty acid profile of the oil obtained are presented in Table 1.

### 2.3. Statistical Design for Determining Oleogel Preparation Conditions

A Box–Behnken response surface methodology (RSM) design was employed to optimize the process of avocado oil oleogel formation. This design was selected over other alternatives, such as the central composite design, because it requires fewer experimental runs while still capturing quadratic effects and interactions among factors, and it avoids extreme operating conditions that could compromise oleogel stability. The independent variables were monoglyceride (MAG) concentration (4, 6, and 8% *w*/*w*), mixing temperature (70, 80, and 90 °C), and heating time (15, 30, and 45 min), with the levels coded as –1, 0, and +1, respectively (summarized in Table 2). These ranges were chosen based on preliminary trials and previous reports indicating that MAGs typically structure oils within 5–20% (*w*/*w*) and require processing temperatures above their melting range (65–85 °C) to promote crystallization and network formation [4,26,27,28,29,30,31,32]. The response variables were oil retention capacity (ORC) and firmness, both of which are critical indicators of oleogel stability and texture and were optimized simultaneously using a desirability function approach. The criteria for optimal conditions were defined as maximizing ORC while maintaining sufficient firmness for structural integrity. A total of 16 experimental runs, including four replicates at the central point, were carried out, and the results are presented in Table 3.

**Figure 2:**
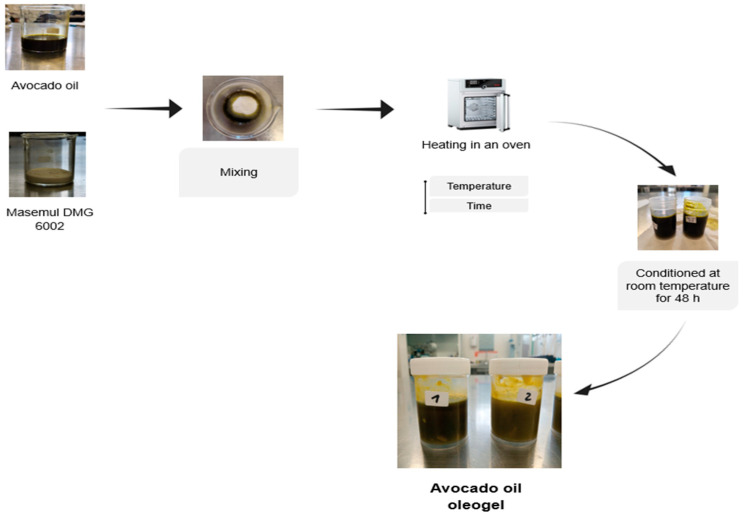
Methodology for obtaining oleogels from avocado oil.

For the preparation of the oleogels, a modification of the methodology proposed by Pérez Monterrosa [27] was used. The structuring agent was weighed according to the concentrations established in the experimental design, using a 100 mL beaker. Subsequently, oil was added to reach a total of 30 g. The mixture was stirred with a metal spatula to disperse the monoglyceride and then heated in a drying oven (MEMMENTH 500h, Hanburg, Germany) at the temperature defined for each trial. To ensure complete dispersion of the structuring agent, manual stirring was performed halfway through the heating time. Once the process was completed, the oleogels were stored in sealed plastic containers (40 mm diameter, 40 mm height) and subjected to a natural cooling process at room temperature for 48 h to perform the analyses. The oleogel preparation process is shown in Figure 2.

#### 2.3.1. Determination of Oil Retention Capacity (ORC)

The oil retention capacity was determined using the Centrifuge Method [7,12]. Approximately 3 g of oleogel were weighed into 50 mL Falcon tubes and centrifuged at 4200× *g* for 30 min at 20 °C. The tubes were then placed upside down on pre-weighed Petri dishes with filter paper for 15 min to drain the released oil. The released oil was weighed, and the oil retention capacity was determined using Equation (1).

(1) $$\mathrm{ORC}\left(\%\right)=\left\{1-\left[\frac{m_{2}-m_{1}}{m}\right]\right\}*100$$

where *m* is the amount of oleogel weighed, *m*_1_ the initial weight of the Petri dish with the filter paper, and *m*_2_ the final weight of the Petri dish with the filter paper and released oil.

#### 2.3.2. Determination of Texture (Firmness)

The firmness of the oleogels was determined through a penetration test using an Acrylic Penetration Probe (P/0.5R) on a texture analyzer (TA.XT Plus Texture Analyzer; Stable Micro Systems, Godalming, UK) equipped with a 30 kg load cell, operated under the following conditions: pre-test speed = 1 mm/s, test speed = 1 mm/s, penetration distance = 10 mm. Firmness values were calculated using the Texture Exponent 32 software (version 4.0.8.0, Stable Micro Systems, Godalming, UK) following established penetration test methodologies commonly applied for the mechanical characterization of monoglyceride-based oleogels and spreadable fat systems [11,30].

### 2.4. Characterization and Stability Analysis of the Optimized Oleogel

#### 2.4.1. Color

Color was determined using a ColorQuest XE spectrophotometer (HunterLAB, Philadelphia, PA, USA) operating in the CIELAB color space, with a D65 illuminant viewing angle and a 10° angle close to the standard observer. The measurement was performed in triplicate for the optimized oleogel and a control sample (oil), using a 1 mm thick plastic cell, ensuring no air bubbles inside. Data were recorded in the L^* (lightness), a^* (+a^*/-a^* for red/green coordinates), and b^* (+b^*/-b^* for yellow/blue coordinates). Additionally, chroma (C*), hue (h*), and the color differences (ΔE, ΔL, Δa*, and Δb*) were calculated using Equation (2) [33].

(2) $$C^{*}=\sqrt{{\left(a^{*}\right)}^{2}+{\left(b^{*}\right)}^{2}}$$

(3) $$h^{*}=\tan^{-1}\left(\frac{b^{*}}{a^{*}}\right)$$

(4) $$∆L=L_{1}-L_{2}$$

(5) $$∆a^{*}={a_{1}}^{*}-{a_{2}}^{*}$$

(6) $$∆b^{*}={b_{1}}^{*}-{b_{2}}^{*}$$

(7) $$∆E=\sqrt{{\left({∆L}^{*}\right)}^{2}+{\left({∆a}^{*}\right)}^{2}+{\left({∆b}^{*}\right)}^{2}}$$

#### 2.4.2. Thermogravimetric and Differential Scanning Calorimetry Analysis (TGA-DSC)

The thermal behavior of avocado oil oleogels was evaluated through thermogravimetric analysis and differential scanning calorimetry (TGA-DSC) using an SDT Q600 V20.9 Build 20 (TA Instruments, New Castle, DE, USA). Approximately 15 mg of oleogel was weighed into a platinum crucible, and the sample was subjected to a temperature range of 27 °C to 150 °C with a heating rate of 5 °C/min in a nitrogen atmosphere at a flow rate of 60 mL/min, following standard procedures for the thermal characterization of lipid-based and structured fat systems [34]. The mass loss of the oil was recorded as the temperature increased, allowing for the identification of thermal decomposition stages and evaluation of its stability. Simultaneously, heat changes associated with phase transitions (such as melting and exothermic/endothermic reactions) were recorded, providing information about the thermal properties of the oleogel.

#### 2.4.3. Determination of Rheological Parameters

Rheological analyses were performed on the oleogel prepared under optimal formulation conditions determined by the response surface methodology using a rheometer (Anton Paar MCR 302, Chicago, IL, USA) with a 25 mm parallel plate geometry and a 1 mm gap. An amplitude sweep (0.01–100%) at 1 Hz frequency and 20 °C was performed to determine the linear viscoelastic region. Additionally, a frequency sweep between 0.01 and 160 Hz and 0.01% shear strain was conducted. Finally, a temperature sweep at 1 Hz and 0.01% shear stress was carried out in a temperature range of 10–100 °C. From these data, the rheological profile of the obtained oleogels was determined [4,12,34,35].

#### 2.4.4. Oxidative Stability

The oxidative stability of pure avocado oil and the oleogel was measured using the 892 Professional Rancimat^®^ method of Metrohm. Approximately 3.5 g of each sample were placed in individual cups, where they were subjected to a temperature of 110 °C and an air flow of 20 L/h. Conductivity in distilled water was monitored to detect volatile oxidation products. The induction time, marking the start of accelerated oxidation, was recorded as an indicator of oxidative stability. The induction times of pure oil and the oleogel were compared to assess the effect of the structuring agent on oxidation resistance [36,37].

#### 2.4.5. Statistical Analysis

The obtained data were analyzed using the response surface methodology (RSM), employing a Box–Behnken experimental design to evaluate the effects of independent variables (concentration, temperature, and processing time) on the oil retention capacity (ORC) and firmness of the oleogel. An analysis of variance (ANOVA) was performed with a significance level of *p* < 0.05 to determine the statistical significance of individual effects and interactions between factors. The results were visualized using response surface plots and Pareto charts, which facilitated the interpretation of main effects and interactions. Statistical analyses were carried out using STATGRAPHICS 18 software (Statgraphics Technologies, Inc., The Plains, VA, USA), and the results were interpreted based on the contribution of each factor to the target properties.

## 3. Results

### 3.1. Statistical Design for Oleogel Preparation Conditions

#### Oil Retention Capacity (ORC) and Texture (Firmness)

**Figure 3:**
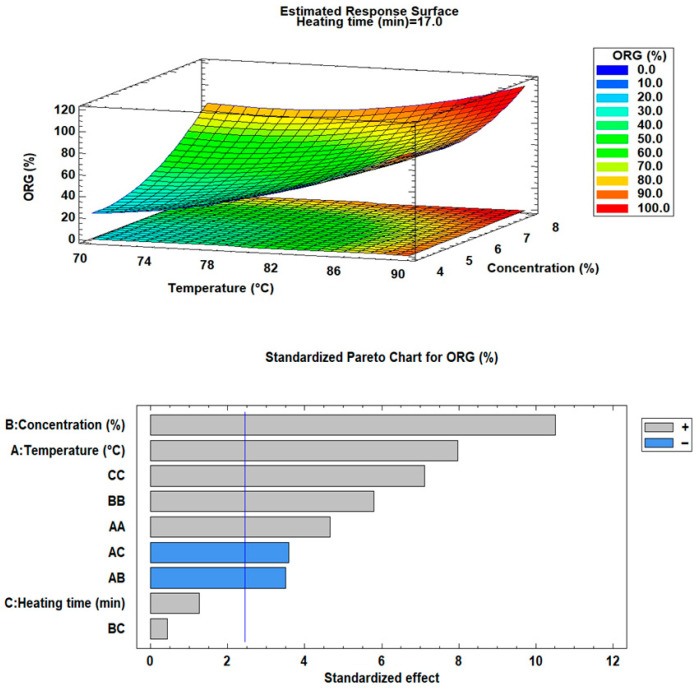
Response surface and Pareto diagram for oil retention capacity (ORC).

**Figure 4:**
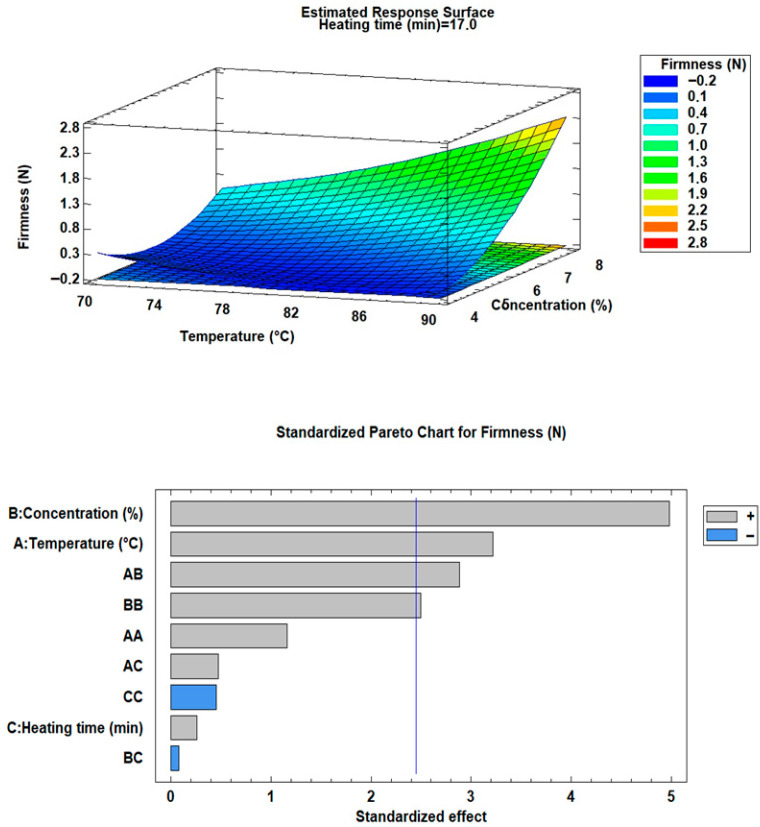
Response surface and Pareto diagram for firmness.

Table 4 presents the results obtained from the experimental design trials, while Figure 3 and Figure 4 illustrate the response surface and Pareto diagrams for ORC and firmness, respectively.

As can be observed, the oleogels exhibited oil retention capacities ranging from 17.80% to 94.70% and firmness values between 0.087 N and 2.764 N. In food applications, high ORC values are desirable since low retention can negatively impact texture, spreadability, and oleogel stability [11].

Firmness values reported as “0.000” correspond to samples in which the penetration force was below the detection limit of the texture analyzer under the selected test conditions. For statistical analysis, these values were treated as zero-force measurements, as they indicate the absence of measurable structural resistance. These data points were included in the ANOVA and response surface model fitting as zero values to reflect the loss of mechanical integrity at those formulation conditions.

In both cases, the ANOVA analysis, supported by response surface and Pareto diagrams, indicated that heating time and its interactions did not have a significant effect (*p* > 0.05) on oil retention capacity (ORC) or gel firmness. This suggests that, within the evaluated ranges, variations in heating time do not substantially influence oil retention or firmness. These findings provide evidence that heating time, within the established limits, does not induce notable structural changes in the gel that could alter its mechanical properties.

### 3.2. Characterization and Stability Analysis of the Oleogel

#### 3.2.1. Selection of the Optimal Formulation

An adjusted regression equation was obtained from the experimental data, incorporating the effects of temperature, concentration, and heating time on oil retention capacity (ORC) (Equation (8)). However, since heating time (Tc, in minutes) showed no significant effect, the model was simplified by excluding the terms associated with this variable (Equation (9)). This not only streamlined the model but also enhanced its interpretability, allowing a focus on the factors that truly influence ORC. The final equation includes only temperature (T, in °C) and concentration (C, in %), facilitating its application in ORC determination calculations.

The decision to base the optimization exclusively on ORC parameters was further supported by the higher goodness of fit indicators (R^2^) obtained for this variable compared to firmness, indicating a more robust and reliable predictive model. From a technological standpoint, ORC represents a primary functional requirement in oleogels, as it directly reflects the ability of the gel network to immobilize the liquid oil phase and prevent phase separation, which is a prerequisite for physical stability in oil systems [34]. Although a multi-response optimization approach was evaluated, the resulting optimal conditions led to reduced values of ORC (65.14%) and firmness (1.26 N), which were considered technologically insufficient for semi-solid fat application. Oleogels with low ORC values are commonly associated with weak or discontinuous networks, which may lead to oil leakage and structural collapse under minimal mechanical stress [11]. Therefore, prioritizing ORC in the optimization ensured the selection of formulations capable of forming a continuous and effective gel network, while firmness was subsequently evaluated as a dependent structural response rather than as a primary optimization constraint.

(8) $$ORC\;(\%)=529.839-0.26625*C-14.7945*T-0.0503333*Ht+4.17031*C^{2}-0.504*C*T\;\;\;\;+0.0416667*C*Ht+0.134412*T^{2}-0.0687333*T*Ht+0.0911611{Ht}^{2}$$

(9) $$ORC\;(\%)=529.839-0.26625*C-14.7945*T+4.17031*C^{2}-0.504*C*T+0.134412*T^{2}$$

Using Equation (8), the combination of temperature and concentration that, according to the model, would theoretically result in an oil retention capacity (ORC) of 100% was identified. The mathematical model determined optimal values of 86 °C for temperature, 7.98% for structuring agent concentration, and 17 min for heating time. Although heating time did not have a significant effect on the analyzed responses, it was retained in the optimization step to define a complete and reproducible set of processing conditions. Consequently, the program-suggested value was adopted as a reference heating time for the process standardization.

#### 3.2.2. Texture Determination (Firmness)

The texture analysis revealed that the obtained oleogel exhibited a firmness value of 1.09 ± 0.094 N.

#### 3.2.3. Color

The color measurement results, expressed in the CIELAB color space (L, a*, and b*), are presented in Table 5. For the oleogel, the low lightness value (L* = 25.35 ± 0.03) indicates a dark appearance, which is consistent with the intrinsic color of avocado oil and the increased light absorption associated with the formation of a structured lipid network. The colorimetric coordinates a* = 0.87 ± 0.03 and b* = 9.95 ± 0.07 suggest a nearly neutral tone with a slight reddish hue and a predominant yellow component, which is expected for the oleogels prepared from avocado oil and monoglycerides [30,34].

The color difference between the oleogel and the oil (ΔE = 11.61) indicates a visually perceptible change, mainly driven by the reduction in lightness (ΔL = −9.39). This behavior has been previously reported in structured lipid systems, where the development of crystalline networks promotes light scattering and reduces reflectance, leading to darker appearances without substantial changes in hue [30,34]. The minimal difference in hue angle (Δh* = 1.15°) further suggests that oleogelation primarily affects color intensity rather than the intrinsic color tone of the oil.

#### 3.2.4. Thermogravimetric Analysis and Differential Scanning Calorimetry (TGA-DSC)

**Figure 5:**
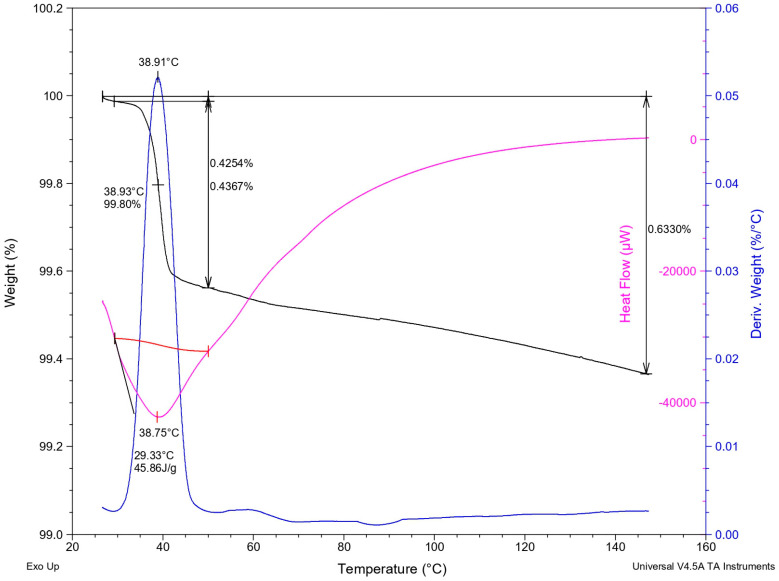
TGA-DSC analysis results for the oleogel with the optimal formulation. The black line corresponds to the remaining mass percentage (weight), the blue line represents the mass loss rate (Deriv. Weight), and the pink line corresponds to the heat flow signal.

The thermal analysis of the oleogel using TGA-DSC revealed an endothermic peak in the range of 27 °C to 45 °C (with a maximum at 38.75 °C), accompanied by a mass loss of 0.43%. This peak suggests a phase transition, likely related to gel disruption, and another peak between 55 °C and 65 °C associated with the melting point of monoglycerides. Additionally, no further thermal events were observed between 45 °C and 150 °C, although a gradual increase in mass loss was recorded, reaching a total of 0.63%. Figure 5 presents the thermal analysis diagram obtained from the measurement.

#### 3.2.5. Determination of Rheological Parameters

**Figure 6:**
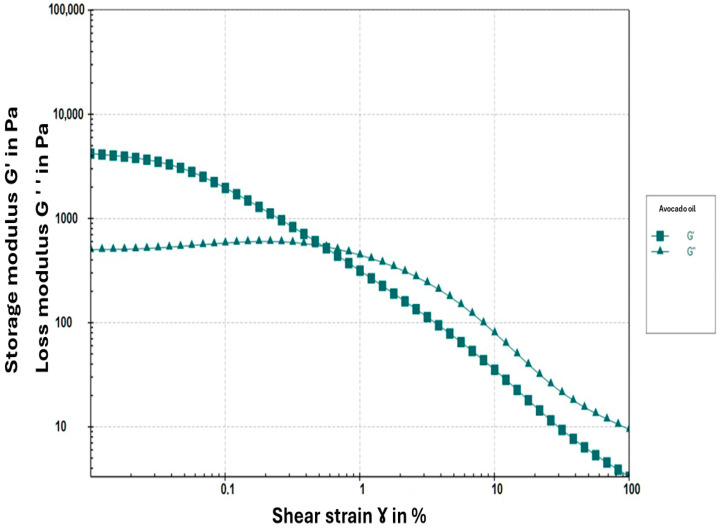
Amplitude sweep of the avocado oleogel as a function of shear strain.

The amplitude sweep analysis (Figure 6) revealed that the oleogel exhibits predominantly elastic behavior, characteristic of a solid-like material. This is evidenced by the storage modulus (G’) being higher than the loss modulus (G″) at low deformations, indicating a greater ability of the gel to store elastic energy. As deformation increases, a crossover point where G’ = G″ is observed, followed by an inversion where G″ exceeds G’, signaling the dominance of viscous properties as the gel structure begins to break down [38]. The gel’s linear viscoelastic region (LVR) was reached at approximately 0.1% strain, suggesting that its structure is relatively weak. This characteristic may limit its application in food matrices requiring higher mechanical stability to withstand greater deformations [38].

**Figure 7:**
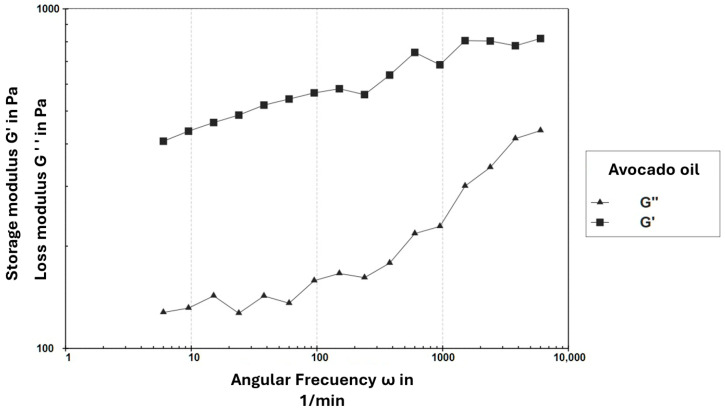
Amplitude sweep of avocado oleogel as a function of angular frequency.

Regarding the frequency sweep analysis (Figure 7), the G′ values remained higher than G″ throughout the entire frequency range evaluated, confirming the predominance of elasticity in the sample. However, both moduli exhibited frequency dependence, indicating that the oleogel responds differently depending on the applied deformation rate. This behavior suggests that the material has some capacity to store elastic energy, although its internal structure may be sensitive to high-frequency stresses.

**Figure 8:**
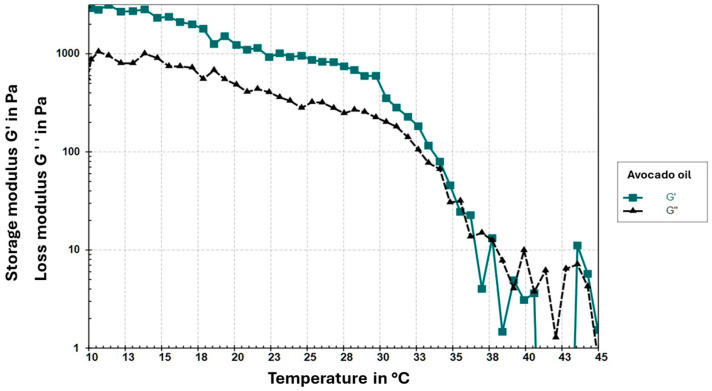
Temperature sweeps of avocado oleogel.

Finally, the temperature sweep (Figure 8) shows that G′ remains higher than G′′ up to approximately 37 °C, indicating structural stability and elasticity within this temperature range. However, around 29 °C, a thermal transition is observed, where both moduli begin to change drastically, reducing the gap between G′ and G′′. This suggests that the gel structure starts to destabilize. This point marks a critical temperature beyond which the oleogel loses its predominantly elastic behavior and its colloidal structure weakens.

The rheological behavior observed in this study is consistent with previous reports on monoglyceride-structured oleogels. Pérez-Monterroza [27], working with avocado oil oleogels, described a viscoelastic profile dominated by the storage modulus (G′ > G″) and a pronounced frequency dependence, both of which match the trends obtained in our formulation. Similarly, Giacomozzi [30] reported that monoglyceride-based oleogels typically exhibit a narrow linear viscoelastic region (LVR < 1%) and a rapid breakdown of the network structure beyond the critical strain, which is consistent with the limited LVR (~0.1%) found in this study. Additional studies by Patel [34] and Ruíz Martínez [35] also describe weak but predominantly elastic networks in food- and pharmaceutical-grade oleogels, where G′ remains higher than G″ as long as the structure remains intact. Furthermore, the thermal transition observed at approximately 29–37 °C aligns with the behavior reported for monoglyceride oleogels subjected to similar heating–cooling conditions [32], suggesting that the mechanical stability of the gel is strongly influenced by the lamellar crystallization of the monoglycerides. Overall, these similarities confirm that the rheological profile of the avocado oil oleogel follows the characteristic pattern of monoglyceride-structured systems, which are dominated by elastic responses but exhibit limited mechanical robustness.

#### 3.2.6. Oxidative Stability

The measurement of oxidative stability using the Rancimat methodology involves determining the induction time. This refers to the period during which an oil or fat sample remains stable before undergoing an accelerated oxidation process. In the comparative analysis between the oil and the oleogel, an induction time of 10.80 h was observed for avocado oil, whereas the oleogel achieved a significantly higher induction time of 41.71 h.

## 4. Discussion

### 4.1. Statistical Design for Oleogel Preparation Conditions

#### Oil Retention Capacity (ORC) and Texture (Firmness)

Conversely, variations in temperature and structuring agent concentration exhibited significant effects (*p* < 0.05), confirming their role as key factors in oil retention capacity and gel firmness enhancement. Higher monoglyceride concentration provides a denser and more stable matrix, as the greater number of crystal aggregates and intermolecular bonds strengthen the network and limit oil migration [19].

Regarding heating temperature, it was found to have a significant effect on both oil retention capacity and firmness. At higher temperatures, monoglycerides dissolve and align within the oil phase, ensuring proper dispersion. Upon cooling, they nucleate and crystallize, forming lamellar structures that propagate into a three-dimensional network. This ordered assembly enhances firmness and limits oil migration [21]. However, when the temperatures exceed critical thresholds (50–70 °C), excessive dissolution or loss of nuclei occurs, delaying crystallization and reducing structural stability. As result, both oil retention and gel firmness are negatively affected [19,20,21].

The statistical models obtained for both analyses demonstrated good fit indicators, as reflected in high R^2^ values. For oil retention capacity (ORC), the R^2^ was 98.08% (adjusted 95.21%), while for firmness, it reached 89.58% (adjusted 73.94%). These results suggest that the main variables (temperature and concentration) largely explain the observed variability in CRA and firmness.

These results are consistent with previous findings on MG-based oleogels and highlight the importance of processing temperature and structurant concentration in directing network formation. For instance, studies on olive oil oleogels, a system with a fatty-acid profile comparable to avocado oil, have reported ORC values between ~80% and ~100% when formulated with 7–10% MGs and processed at temperatures between 70 and 90 °C [11,31]. These studies similarly emphasize that higher MG concentrations promote denser crystalline aggregates that effectively immobilize the oil, while insufficient crystallization at either excessively low or overly high temperatures reduces oil entrapment. Giacomozzi [2] also observed that MG-based oleogels display improved oil binding when processed above the complete melting range of MGs (≈65–85 °C), followed by controlled cooling, conditions aligned with those optimized in the present study. In avocado oil systems, Pérez-Monterroza [27] described that monoglyceride crystallization produces lamellar structures similar to those in olive oil oleogels, although their work did not quantify ORC. Therefore, the trends observed in our formulations (increasing ORC with higher MG concentration and optimal temperatures around the MG melting range) are in agreement with the crystallization mechanisms described for similar monoglyceride-structured matrices. Furthermore, the variability in ORC observed across the experimental matrix (17.8–94.7%) reflects the sensitivity of avocado oil to processing conditions, likely influenced by its specific unsaturated lipid profile and minor polar compounds, which may affect nucleation kinetics compared to other oils.

### 4.2. Characterization and Stability Analysis of the Oleogel

#### 4.2.1. Selection of the Optimal Formulation

After defining the optimal parameters, the oleogel was prepared, and its oil retention capacity was measured. A discrepancy was observed between the estimated theoretical value (ORC = 100%) and the actual performance (ORC = 85.95 ± 1.96%). This deviation falls within the expected perdition error associated with response surface methodology (RSM) models, which provide an approximation of systems behavior based on fitted polynomial equations and are subject to experimental variability and model assumptions [39]. In addition to the intrinsic limitations of the model, this difference may be related to the crystallization conditions of the gels, which were formed at room temperature, as well as potential variations in the distribution of the structuring agent that could have influenced the oleogel’s structure. Previous studies have shown that ORC can be affected by the crystallization temperature [11,31]. Additionally, authors such as Chen and Pérez-Monteroza [19,27] have demonstrated that component distribution and network formation kinetics are crucial for achieving optimal properties in oleogels.

At present, research on avocado oil oleogels structured with monoglycerides primarily focuses on gel morphology and rheology [27,38], with no data reported on ORC for direct comparison. Therefore, as a reference matrix, oleogels derived from olive oil and monoglycerides were used, given their similar fatty acid profile to avocado oil. Ögütcü and Yimaz [11] developed olive oil oleogels using monoglycerides at concentrations of 7% and 10%, obtaining ORC values of 80.72% and 99.87%, respectively.

#### 4.2.2. Texture Determination (Firmness)

Comparing the results with previous studies, Ögütcü and Yılmaz [11] reported firmness values ranging from 0.74 to 2.94 N for olive oil oleogels structured with 7% and 10% monoglycerides, indicating that monoglyceride concentration plays a key role in determining the mechanical strength of the gel network. In the same study, a commercial margarine exhibited a firmness of 0.66 N, highlighting the overlap between the textural properties of MG-based oleogels and conventional soft fat products [11]. Similarly, Zampouni et al. [31] reported a firmness of approximately 1.2 N for an olive oil oleogel containing 10% monoglycerides, which is consistent with oleogels designed for spreadable fat applications. The firmness obtained for the optimized avocado oil oleogel in the present study falls within this reported range [11,31], indicating that the developed system achieves a balance between structural integrity and spreadability. This level of firmness is considered desirable for applications such as spreads and soft fat replacers, where adequate mechanical strength is required to ensure oil immobilization while maintaining acceptable mouthfeel and processability [11,31].

#### 4.2.3. Color

**Figure 9:**
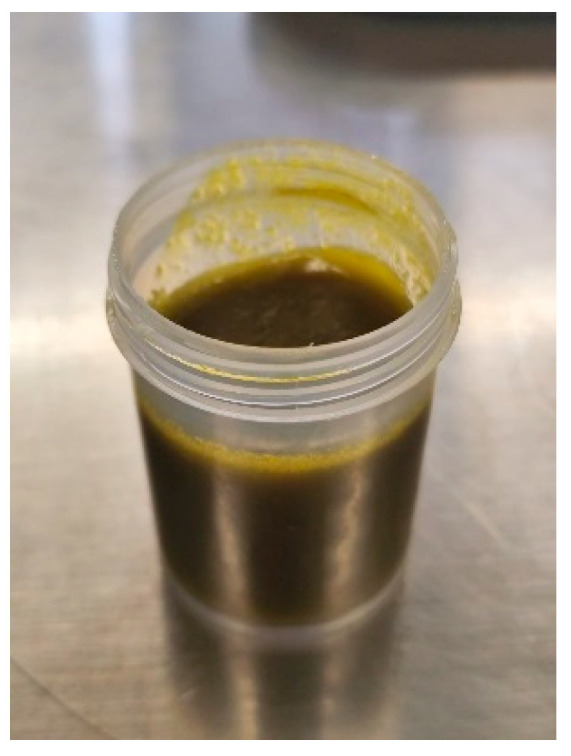
Oleogel prepared under the model’s optimal conditions.

In relation with these results, the hue angle (h* = 84.80 ± 0.170°) places the color within the yellow-green range, with a stronger inclination toward yellow, which can be attributed to the presence of natural pigments such as carotenoids in the oil [31]. The chroma value (C* = 9.98 ± 0.06) indicates moderate saturation, which may be desirable in products where a natural rather than artificial appearance is preferred. Figure 9 shows the obtained oleogel.

#### 4.2.4. Thermogravimetric Analysis and Differential Scanning Calorimetry (TGA-DSC)

The minor mass loss observed within this temperature range may be attributed to the evaporation of trace volatile compounds or residual low-molecular-weight components trapped within the oleogel network [39,40]. The absence of abrupt mass losses or multiple degradation steps indicates the oleogel matrix remains thermally stable and does not undergo significant decomposition below 150 °C. This behavior is consistent with previous reports on monoglyceride-based oleogels, where thermal events detected in the 30–40 °C range are associated with melting or reorganization of laminar crystalline structures rather than chemical degradation of the lipid phase [30,32]. Overall, these results confirm that the thermal response of the oleogels is governed by physical transitions of the structuring network, supporting its structural robustness and suitability for food applications requiring moderate thermal resistance, such as spreads and soft fat systems, where processing temperatures typically remain below this threshold [30,32].

#### 4.2.5. Determination of Rheological Parameters

The rheological behavior observed in this study is consistent with previous reports on monoglyceride-structured oleogels. Pérez-Monterroza [27], working with avocado oil oleogels, described a viscoelastic profile dominated by the storage modulus (G′ > G″) and a pronounced frequency dependence, both of which match the trends obtained in our formulation. Similarly, Giacomozzi [30] reported that monoglyceride-based oleogels typically exhibit a narrow linear viscoelastic region (LVR < 1%) and a rapid breakdown of the network structure beyond the critical strain, which is consistent with the limited LVR (~0.1%) found in this study. Additional studies by Patel [34] and Ruíz Martínez [35] also describe weak but predominantly elastic networks in food- and pharmaceutical-grade oleogels, where G′ remains higher than G″ as long as the structure remains intact.

From an application perspective, the narrow LVR and limited mechanical robustness observed suggest that the oleogel network may be sensitive to moderate deformations, potentially restricting its direct use in food systems subjected to high mechanical stress, such as laminated bakery fats or products requiring intensive mixing [34]. However, similar rheological profiles have been reported for oleogels intended for low-deformation applications, such as spreads or soft fat replacers, where elastic dominance and sufficient oil immobilization are more critical than high mechanical strength [30,34]. Previous studies have also demonstrated that the mechanical robustness of monoglyceride-based oleogels can be enhanced through formulation strategies such as increasing gelator concentration or modifying crystallization conditions, which promote denser lamellar crystal networks and improved resistance to deformation [30,32].

Furthermore, the thermal transition observed at approximately 29–37 °C aligns with the behavior reported for monoglyceride oleogels subjected to similar heating–cooling conditions [32], suggesting that the mechanical stability of the gel is strongly influenced by the lamellar crystallization of the monoglycerides. Overall, these similarities confirm that the rheological profile of the avocado oil oleogel follows the characteristic pattern of monoglyceride-structured systems, which are dominated by elastic responses but exhibit limited mechanical robustness.

#### 4.2.6. Oxidative Stability

The oxidative stability results obtained in this study align with trends reported for other oleogels structured with monoglycerides and similar agents. Several authors have shown that the formation of a three-dimensional network restricts oxygen diffusion and reduces the mobility of unsaturated lipids, thereby delaying the onset of oxidation. For example, Fayaz [4] reported a three- to four-fold increase in induction time for pomegranate oil oleogels structured with monoglycerides, while Shuai [23] observed a marked improvement in the oxidative resistance of macadamia oil oleogels formulated with MG. Likewise, Giacomozzi [2] demonstrated that MG-based oleogels consistently exhibit superior oxidative stability compared to the corresponding liquid oils due to reduced radical propagation within the gel matrix. Similar protective effects have also been described in oleogels structured with polysaccharides or waxes, which similarly act as physical barriers limiting access of pro-oxidant species [6,7]. In this context, the four-fold increase in induction time observed for the avocado oil oleogel (41.71 H vs. 10.80 h in the pure oil) is consistent with the antioxidative behavior expected from structured lipid systems and highlights the ability of MG crystallization to shield the oil phase from oxidative degradation.

## 5. Conclusions

The results confirm that monoglyceride concentration and temperature are the main variables governing the structuring of avocado oil oleogels, while heating time has no significant impact. This allows the process to be simplified by eliminating prolonged heating, reducing production costs without affecting product quality. The optimized oleogels showed high oil retention, firmness, and oxidative stability, supporting their potential use as healthier substitutes for conventional solid fats in food and cosmetic applications.

In addition to their functional performance, the use of avocado oil offers an added advantage, as it provides an alternative use for non-export-grade fruit, contributing to the reduction in postharvest losses and the valorization of surplus production. Rheological and thermal analyses revealed predominantly elastic behavior, good oxidative resistance, and stable performance within the evaluated temperature range. However, the moderate gel strength observed suggests that further formulation adjustments may be required for applications demanding higher mechanical stability. Compared with commercial margarine and other vegetable oil-based oleogels, the avocado oil–monoglyceride system demonstrated promising firmness and stability, reinforcing its applicability and highlighting the importance of controlling formulation and processing conditions to ensure consistent performance.

## Figures and Tables

**Table 1:** Quality parameters and fatty acid profile of the oil obtained.

Parameter	Value	Methodology
Palmitic acid (C16:0) (%)	26.08	AOAC ce 1i-07
Palmitoleic acid (C16:1) (%)	0.10	
Oleic acid (C18:1) (%)	45.69	
Linoleic acid (C18:2) (%)	13.85	
Linolenic acid (C18:3) (%)	0.99	
L*	34.74 ± 0.32	Methodology proposed by Sanchez–Reinozo [26]
a*	0.34 ± 0.13	
b*	3.15 ± 0.16	
Density (g/mL)	0.920 ± 0.001	AOAC 920.212
Refractive index	1.4620 ± 0.0002	AOAC 921.08
Peroxide value (meq O_2_/(Kg oil)	13.322 ± 0.324	AOAC 965.33
*p*—Anisidine value	1.845 ± 0.337	AOAC cd 18-90
Acid value (% oleic acid)	0.110 ± 0.005	

**Table 2:** Levels coded in the Box–Behnken experimental design.

Variable	Low (−1)	Medium (0)	High (1)
Mixing Temperature (°C)	70.0	80.0	90.0
Heating time (min)	15.0	30.0	45.0
Concentration (% *w*/*w*)	4.0	6.0	8.0

**Table 3:** Formulations used for the experimental design.

Trial	Temperature (°C)	Concentration (%)	Heating Time (min)
1	70	4	30
2	70	6	45
3	70	6	15
4	70	8	30
5	80	4	15
6	80	4	45
7	80	8	45
8	80	8	15
9	80	6	30
10	80	6	30
11	80	6	30
12	80	6	30
13	90	6	45
14	90	6	15
15	90	4	30
16	90	8	30

**Table 4:** Results of oil retention capacity and firmness determination.

Trial	ORC (%)	Firmness (N)
1	17.80	0.087
2	69.19	0.090
3	38.64	0.000
4	82.33	0.568
5	53.85	0.098
6	51.66	0.000
7	95.45	1.043
8	92.64	1.200
9	35.34	0.000
10	44.08	0.000
11	38.22	0.814
12	27.19	0.000
13	81.06	0.849
14	91.75	0.404
15	70.49	0.129
16	94.70	2.764

The value “0.000” reported in Table 4 corresponds to samples for which the resistance to penetration was below the detection limit of the probe under the selected test conditions.

**Table 5:** CIELAB colorimetric coordinates.

Sample	L*	a*	b*
Oil	34.74 ± 0.32	0.34 ± 0.13	3.15 ± 0.16
Oleogel	25.35 ± 0.03	0.87 ± 0.03	9.95 ± 0.07

## Data Availability

The original contributions presented in this study are included in the article. Further inquiries can be directed to the corresponding author.

## Abbreviations

The following abbreviations are used in this manuscript:

ORC	Oil Retention Capacity
MGs	Monoglycerides
RSM	Response Surface Methodology
TGA-DSC	Thermogravimetric and Differential Scanning Calorimetry Analysis
ANOVA	Analysis of Variance
